# Preoperative assessment of skeletal muscle mass during magnetic resonance enterography in patients with Crohn’s disease

**DOI:** 10.1007/s13304-020-00790-x

**Published:** 2020-05-14

**Authors:** V. Celentano, L. Kamil-Mustafa, R. Beable, C. Ball, K. G. Flashman, Z. Jennings, D. P. O’ Leary, A. Higginson, S. Luxton

**Affiliations:** 1 Queen Alexandra Hospital – Portsmouth Hospitals NHS Trust, Portsmouth, UK; 2 University of Portsmouth, Portsmouth, UK

**Keywords:** Crohn’s disease, Psoas muscle, Colorectal surgery, Sarcopenia, Magnetic resonance imaging, Inflammatory bowel disease

## Abstract

Measurement of the psoas muscle area has been applied to estimate lean muscle mass as a surrogate marker of sarcopenia, but there is a paucity of evidence regarding the influence of sarcopenia on clinical outcomes following inflammatory bowel disease surgery. The aim of this study was to evaluate the association between MRI enterography defined sarcopenia and postoperative complications in patients undergoing elective ileocaecal resection for Crohn’s disease. To obtain cross sectional area measurement of the psoas muscle, the freehand area tool was used to trace the margin of each psoas muscle at the level of L4, with the sum recorded as Total Psoas Area (TPA). The total cross sectional muscle area of the abdominal wall was recorded as Skeletal Muscle Area (SMA), while myosteatosis was measured by normalising the psoas muscle intensity with the mean intensity of the cerebrospinal fluid. The primary outcome was the incidence of 30-day postoperative complications in patients in the lowest quartile of TPA and SMA. 31 patients were included and ten patients (32.25%) developed postoperative complications within 30 days of surgery. The cut-off values for the lowest quartile for TPA were 11.93 cm^2^ in men and 9.77 cm^2^ in women, including a total of 8 patients (25.8%) with 5 patients in this group (62.5%) developing postoperative complications and 3 patients (37.5%) Clavien-Dindo class ≥ 3 complications. The cut-off values for the lowest quartile for SMA were 73.49 cm^2^ in men and 65.85 cm^2^ in women, with 4 patients out of 8 (50%) developing postoperative complications. Psoas muscle cross sectional area and skeletal mass area can be estimated on Magnetic Resonance Enterography as surrogate markers of sarcopenia with high inter-observer agreement.

## Introduction

Measurement of the psoas muscle area has been increasingly applied to estimate lean muscle mass as a surrogate marker of sarcopenia, which is a reduction in lean body mass and function [1]. Computed tomography (CT) assessment of the psoas muscle cross sectional area has been shown to correlate with adverse postoperative outcomes following gastrointestinal, endocrine, urological, and transplant surgery [2, 3] but there is a paucity of evidence regarding the influence of sarcopenia on clinical outcomes following inflammatory bowel disease (IBD) surgery. Loss of lean muscle mass estimated by measuring the cross-sectional area and density of the psoas muscle preoperatively has been associated with a significantly increased risk of developing major complications following resection for colorectal cancer especially anastomotic leak [4]. A number of studies have used a radiological assessment of fat and skeletal muscle compartments to predict disease activity and progression in Crohn’s disease (CD) with lower muscle attenuation and a high visceral fat index having been associated with more severe phenotypes [5] and L3 skeletal muscle area being demonstrated a prognostic factor for intestinal resection in CD patients [6]. Sarcopenia has also been suggested to be a novel predictor of need for medical or surgical rescue therapy in acute severe ulcerative colitis patients [7]. However, the role of radiologically assessed sarcopenia in predicting postoperative outcomes in CD patients remains a debatable yet growing topic [8, 9].

CD requires surgical treatment in up to 80% of patients. Surgery for CD carries high risks of complications including wound infections, anastomotic leak and intraabdominal sepsis, which are made more likely by immune suppression, malnutrition and penetrating or recurrent disease. Ileo-caecal resections for CD have a similar complication profile to those undergoing a similar surgery for cancer [10], despite CD patients being around 30 years younger and with less co-morbidity. Patients with CD also often report weight loss and malnutrition [11]. Changes in body composition have been described in patients with CD with prevalence of low lean mass reported in up to 28% of patients [12]. Lean mass deficits have been associated with demonstrable morbidity and mortality [13], with IBD patients being particularly at risk in view of the associated chronic inflammation [14].

Patients with CD require a multidisciplinary approach and when surgery is undertaken it should be carried out by a colorectal surgeon who is a core member of the IBD multidisciplinary team [15] with audited outcomes (stoma rate, complications, re-interventions and mortality) [16]. Common indications for surgery include abscesses, complex internal fistulae and fibrostenotic strictures. Many imaging modalities are available to plan the surgical treatment and include a combination of abdominal ultrasound scan (USS), CT and magnetic resonance imaging (MRI). In view of the chronic nature of CD patients often require repeated radiological investigations, so imaging modalities that do not involve ionizing radiation should be used whenever possible [17]. Incorporating a radiological assessment of sarcopenia into the preoperative work up of CD may identify patients most at risk of postoperative complications, improving preoperative counselling, identifying those who may benefit from prehabilitation interventions and perhaps influencing operative strategy such as avoiding a primary anastomosis [18].

The aim of this pilot study was to evaluate the association between MRI enterography (MRE) defined sarcopenia and postoperative complications in patients undergoing elective ileocaecal resection for CD.

## Methods

### Study setting

This retrospective observational study included all patients having elective ileocaecal resection for primary CD during a 2 years period from 1st of March 2017 to 28th of February 2019 at Queen Alexandra Hospital (Portsmouth, United Kingdom). Patients undergoing emergency operations or surgery for CD recurrence were excluded. The study was designed according to the STROBE checklist [19]. The indication for surgical resection was discussed at dedicated IBD multidisciplinary team meetings (MDT) involving gastroenterologists, colorectal surgeons, radiologists and pathologists. Preoperative assessment included colonoscopy, MRE and intestinal ultrasound.

### Data collection

Preoperative, operative and postoperative data were recorded prospectively for each patient on a dedicated database. Preoperative parameters included age, sex, body mass index (BMI), comorbidities, American Society of Anaesthesiologists (ASA) status, albumin and haemoglobin concentration, smoking status, weight loss, indication for surgery, preoperative medical therapy and Montreal classification. Operative data included surgical approach, operating time, intraoperative complications, estimated operative blood loss, conversion rate, reason for conversion and use of temporary ileostomy. Postoperative data included length of hospitals stay (LOS) and postoperative complications according to the Dindo-Clavien classification [20].

### MRE protocol and image analysis

Preoperative MRE scans were performed at Queen Alexandra Hospital on either a GE or Siemens 1.5T MRI scanner, with a GE 3T scanner occasionally utilised as the departmental requirements dictated. For the purposes of the study, the axial T2 weighted (T2-SSFSE) sequences were assessed. Three readings were independently performed by a radiologist, a surgeon and an advanced practitioner radiographer in MRI, blinded to the surgical outcomes. An image slice at the level of the superior endplate of L4 was selected for assessment by the radiologist, and the slice number was recorded to enable direct comparison with both the surgical and radiographer readers.

**Fig. 1:**
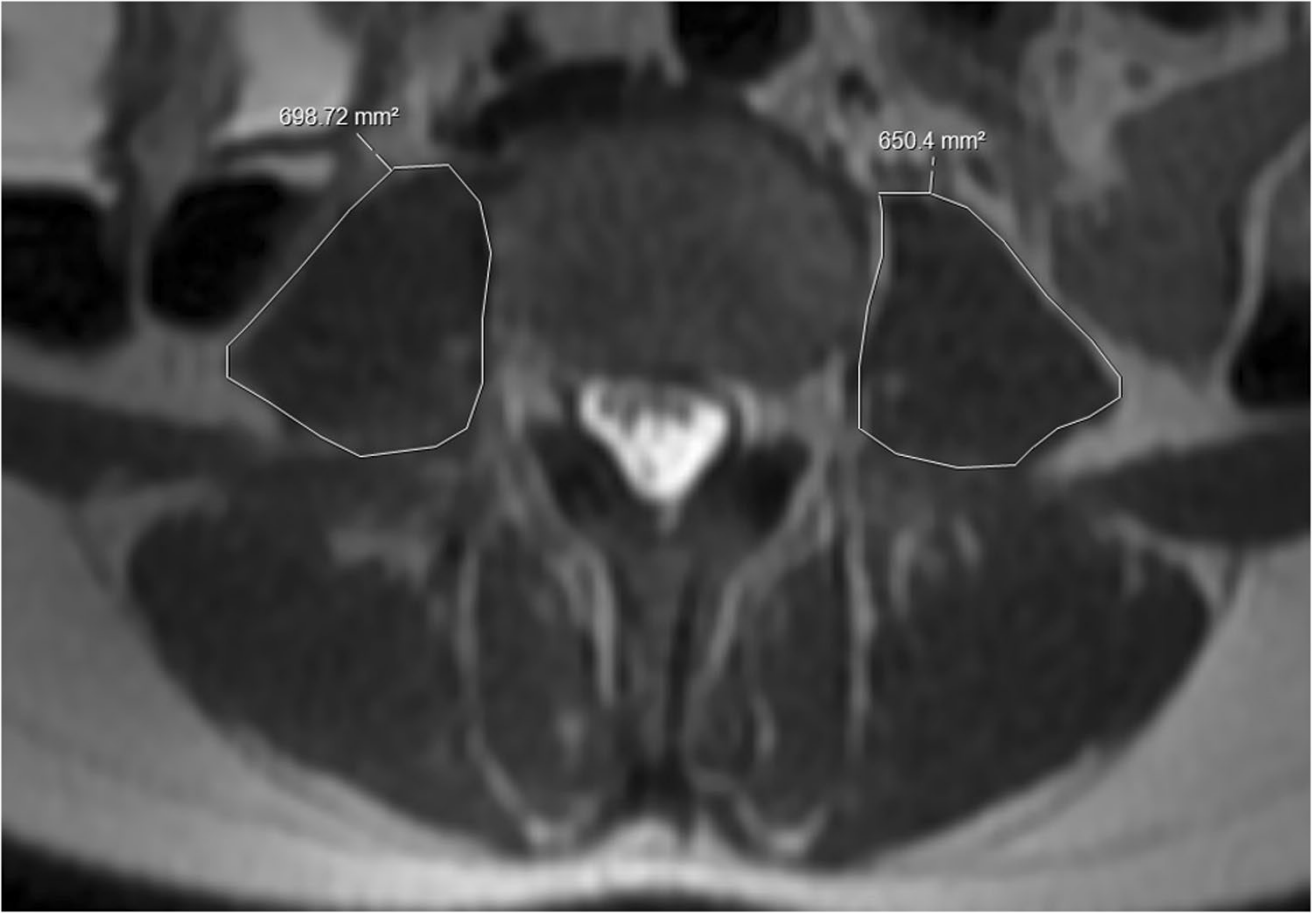
Cross sectional area of both psoas muscles measured at the level of the superior endplate of L4 on axial T2 weighed imaging

**Fig. 2:**
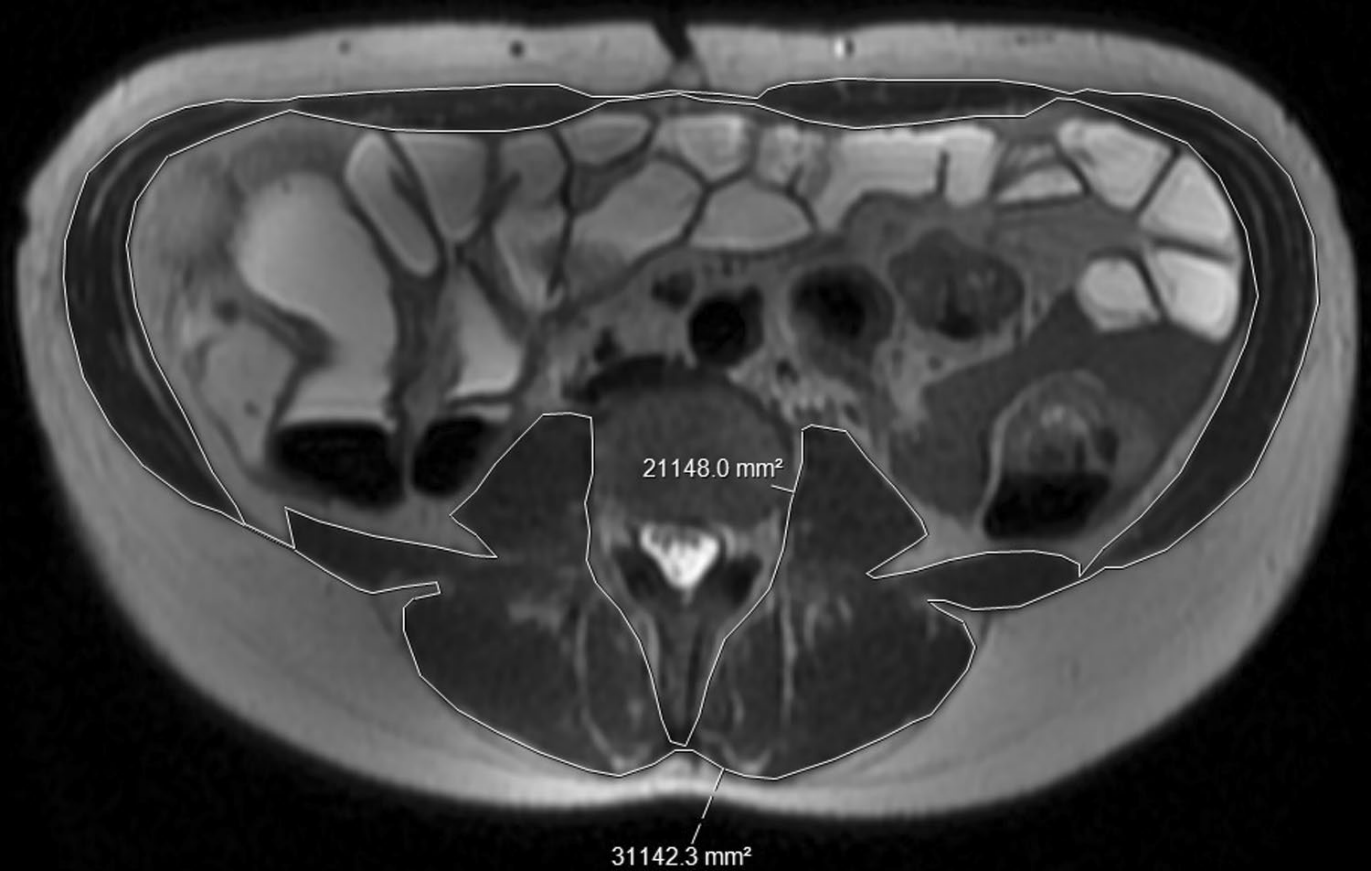
Cross sectional total muscle area at the level of the superior endplate of L4 on axial T2 weighed imaging, calculated by subtracting the inner area from the outer muscle area

Analysis was performed using the measuring software available on the IDS7 Sectra picture archiving and communication system (PACS) workstation (version 19.3.3). Cross sectional area is a relatively quick measurement able to be performed using most modern PACS software programs, and is considered a valid marker for sarcopenia [21]. To obtain cross sectional area measurement of the psoas muscle, the freehand area tool was used to trace around the margin of each psoas muscle, to include the intercellular adipose tissue (Fig. 1). Measurement of each psoas was performed at the same level, with the sum of the two muscles recorded as Total Psoas Area (TPA). To assess the total cross sectional muscle area of the abdominal wall at this level (skeletal muscle area—SMA), two separate measurements were obtained using the same tool, to measure the outer margin of the muscle groups, and the inner margin, with the free hand tool enabling the exclusion of the vertebra (Fig. 2). By subtracting the second measurement from the first, the total cross sectional area of all muscle groups was obtained.

TPA and SMA were normalised to the body surface area of the patient, calculated according to the Mosteller formula which takes the square root of the height expressed in centimetres multiplied by the weight expressed in kilograms, divided by 3600 [22]. The Skeletal Muscle Index (SMI) was obtained by correcting the SMA for height: SMA (cm^2^)/height (m) ^2^ [23].

**Fig. 3:**
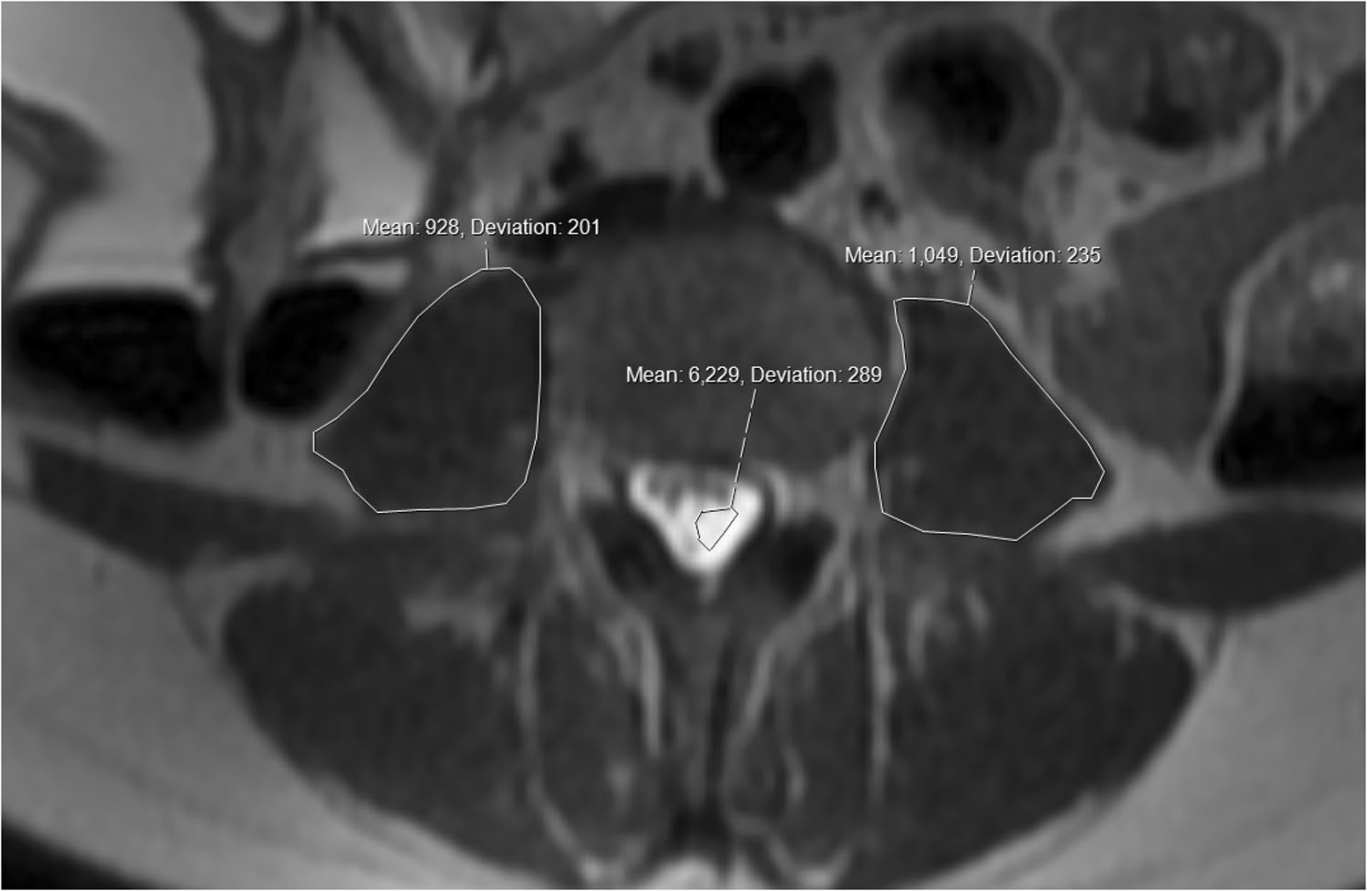
Free hand region of interest (ROI) area measurement of the signal intensity of both psoas muscles, and the signal intensity of the cerebrospinal fluid at the same level

Assessment for myosteatosis on MRI is slightly more complex than measuring atomic density on CT. Accurate assessment of inter and intracellular fat can be obtained using additional sequences including MR spectroscopy or with chemical shift sequences [24], which are generally considered a more accurate method. These sequences are not performed routinely given the time restraints on MRI scanners, and were therefore unavailable in this retrospective study. Fat returns a higher signal on T2 weighted imaging than skeletal muscle, and it is proposed that an increase in the signal intensity of the muscle is likely due to increased inter and intra muscular fat content. Given the variability in scanning parameters between different scanners, as well as the differences in scaling between patients, it was not possible to simply measure the signal intensity. An internal standard was therefore utilised, in this case the mean signal intensity of the cerebrospinal fluid (CSF), and a ratio was measured. This has been previously shown to be a valid representative measure of myosteatosis [25]. The same axial T2 weighted imaging was used for this assessment, with the musculature first assessed on the separate fat suppressed sequences to ensure that any elevated signal on the straight T2 sequence was not secondary to intramuscular oedema (which would also return a higher T2 signal). The signal intensity was obtained for both psoas muscles using the free hand tool on the IDS7 Sectra PACS workstation, to encompass the entirety of each psoas muscle (including intercellular adipose tissue). The mean of the two measurements was obtained. The CSF signal intensity was also measured, excluding any of the cauda equina nerve rootlets, ideally at the same level (Fig. 3). However, if there were interfering factors (such as spinal canal stenosis or significant CSF flow void artefact), a different level was selected from the same axial sequence.

Since there are no published cut-offs for MRE based measurements of TPA and SMA we determined gender-specific cut-off values based on the lowest quartile [26], as previously reported. As higher signal intensity is associated with a higher presence of intramuscular fat, the highest quartile was chosen to indicate sarcopenia when evaluated using the psoas muscle intensity to CSF ratio.

For the diagnosis of sarcopenia, the SMI cut-off values in a healthy, younger population (20–60 years old) have been previously recommended for CT scan imaging, which are 43.1 cm^2^/m^2^ in men and 32.7 cm^2^/m^2^ in women [23]. However, as our study focused on patients with CD and the measurements were achieved via MRI imaging, also for the SMI parameter the lowest quartile cut-off was adopted, as considered more appropriate.

### Surgical procedure

Surgery was performed with a laparoscopic approach with full mobilisation of the terminal ileum and right colon and extracorporeal division of the mesentery and anastomosis [27]. No intra-abdominal drains were left routinely. Postoperatively patients were managed according to an enhanced recovery protocol.

### Primary and secondary outcomes

The primary outcome was the incidence of 30-day postoperative complications in patients with sarcopenia, defined as patients in the lowest quartile of TPA and SMA. Secondary outcome was inter-observer variability of psoas muscle and abdominal wall measurement.

### Statistical analysis

Categorical variables are presented as frequency or percentage and were compared with the use of the Chi square test or Fisher’s exact test, as appropriate. Continuous variables are presented as mean (± standard deviation) or median (range) and were compared with the use of Student’s t test. The Mann–Whitney *U* test was used for continuous, not normally distributed outcomes. To assess agreement between measurements of a continuous variable across observers, the intra-class correlation coefficient (ICC) was used. The ICC provides a single measure of the extent of agreement ranging from 0 to 1, with high values indicating high correlation [28, 29]. Statistical analysis was performed by using the Statistical Package for Social Sciences (SPSS version 16.0; SPSS, Chicago, IL, USA) and GraphPad Prism version 8.0.2 for Windows, (GraphPad Software, La Jolla California USA, www.graphpad.com). All reported *p* values were two-tailed, and *p* values of less than 0.05 were considered to indicate statistical significance.

### Ethics

The study is conducted in accordance with the principles of the Declaration of Helsinki and ‘good clinical practice’ guidelines. The study was registered as an audit and no sensitive patients’ data were collected, therefore ethical approval was not needed.

## Results

31 patients were included. Preoperative data and postoperative outcomes are summarised in Table 1. All procedures were performed laparoscopically with no conversions to open surgery. A temporary ileostomy was fashioned in 2 patients (6.45%) and in both cases this had been already reversed at the 12 months follow up visit.

**Table 1:** Patients’ baseline characteristics and short term outcomes

Included patients (*n*)	31
Age (years)	46 (range 19–72)
Sex	Male 14 (45.2%) Female 17 (54.8%)
BMI	24.4 (21.8–28)
Previous abdominal surgery	11 (35.48%)
Penetrating phenotype CD	12 (38.7%)
Preoperative steroids treatment	4 (12.9%)
Preoperative anti-TNF treatment	8 (25.8%)
Hypoalbuminemia*	7 (22.58%)
Anaemia**	8 (25.8%)
Weight loss > 10%	6 (19.35%)
Operating time (minutes)	190 (180–240)
Temporary ileostomy	2 (6.45%)
Blood loss (mls)	75 (50–200)
LOS	7 (6-9)
30 day complications	10 (32.25%)
30 day Clavien-Dindo grade ≥ 3	5 (16.12%)
Readmissions	1 (3.22%)
Reoperations	1 (3.22%)

*n* number, *BMI* body mass index, *CD* Crohn’s disease, *TNF* tumour necrosis factor, *mls* millilitres, *LOS* length of hospital stay

*Preoperative albumin level < 35 g/L

**Preoperative haemoglobin level < 12 g/dL

### Postoperative complications

Ten patients (32.25%) developed postoperative complications within 30 days of surgery. These were 2 chest infections, 1 wound infection, 1 bleeding and 6 intra-abdominal collections. The patient with bleeding required laparotomy and washout whilst 4 collections required radiological guided drainage for a total of 5 Clavien Dindo class 3 or higher complications (16.12%), 1 patient was readmitted (3.22%) for non-specific abdominal pain.

### Total psoas area (TPA) and skeletal muscles area (SMA) measurements

TPA was 11.79 cm^2^ (range 8.92–18.01), while SMA was 71.41 cm^2^ (range 51.94–92.9).

**Fig. 4:**
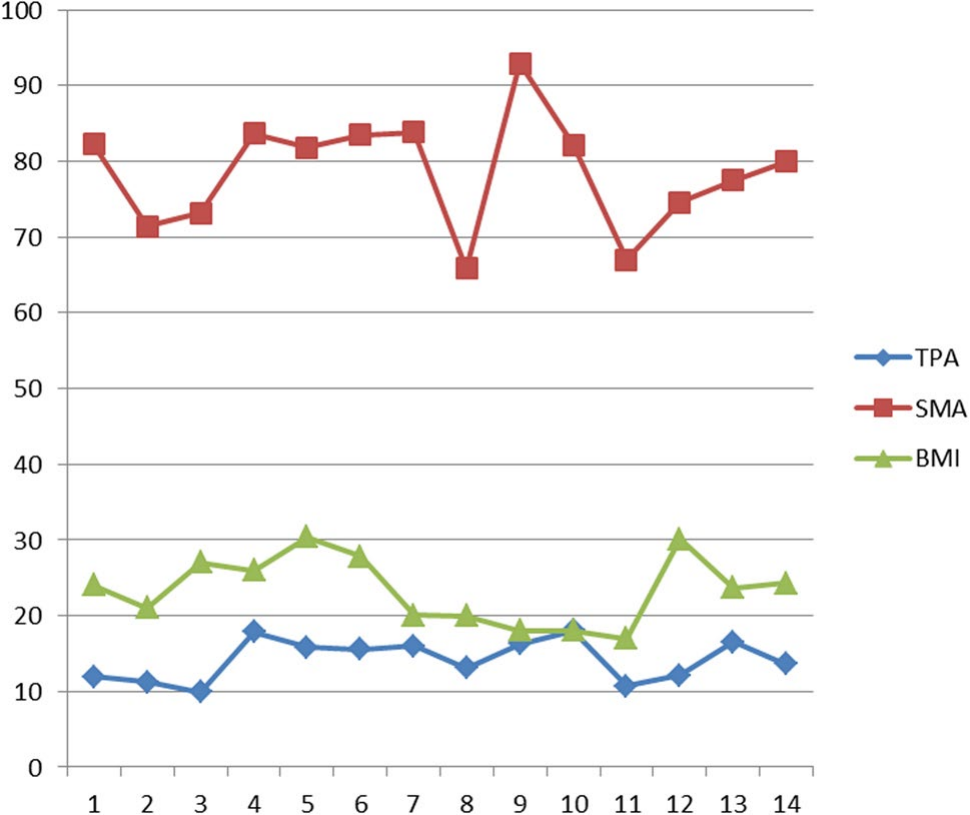
Absolute values of total psoas area (TPA), total abdominal muscle area (SMA) and body mass index (BMI) in the included 14 male patients

**Fig. 5:**
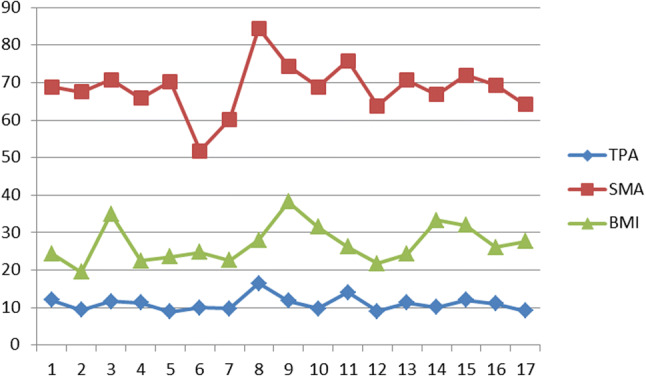
Absolute values of total psoas area (TPA), skeletal muscle area (SMA) and body mass index (BMI) in the included 17 female patients

TPA correlated with SMA (*p* < 0.0001) but not with age, BMI or preoperative albumin and Haemoglobin levels (Figs. 4 and 5).

The cut-off values for the lowest quartile for TPA were 11.93 cm^2^ in men and 9.77 cm^2^ in women, including a total of 8 patients (25.8%) with 5 patients in this group (62.5%) developing postoperative complications (*p* = 0.46) and 3 patients (37.5%) Clavien-Dindo class ≥ 3 complications (*p* = 0.25).

The cut-off values for the lowest quartile for SMA were 73.49 cm^2^ in men and 65.85 cm^2^ in women. A SMA below the cut-off value was present in 8 patients (25.8%) with 4 patients out of 8 (50%) developing postoperative complications (*p* 0.93) and 3 patients (37.3%) developing grade ≥ 3 Clavien-Dindo complication (*p* = 0.25). There was no increased prevalence of preoperative steroids and anti-TNF treatment, penetrating phenotype of disease, hypoalbuminemia and anaemia in the group of patients with MRE defined sarcopenia.

Median SMI was 47.06 cm^2^ (range 37.27–60.41). The cut-off values for the lowest quartile for SMI were 48.88 cm^2^ in men and 39.67 cm^2^ in women. A SMI value below the cut-off was present in 7 patients (22.5%) with 2 patients developing postoperative complications (28.5%) which were in both cases grade ≥ 3 Clavien-Dindo (28.5%).

### Psoas intensity to CSF ratio

The cut-off value for the highest quartile of the psoas muscle intensity to CSF ratio was 0.107 with 8 patients (25.8%) above the cut-off value. There were a total of 4 complications (50%) with 3 patients out of 8 having Clavien-Dindo grade ≥ 3 (37.5%). There was no correlation with TPA, SMA and BMI.

### Prevalence of MRE defined Sarcopenia in patients who developed postoperative complications

**Fig. 6:**
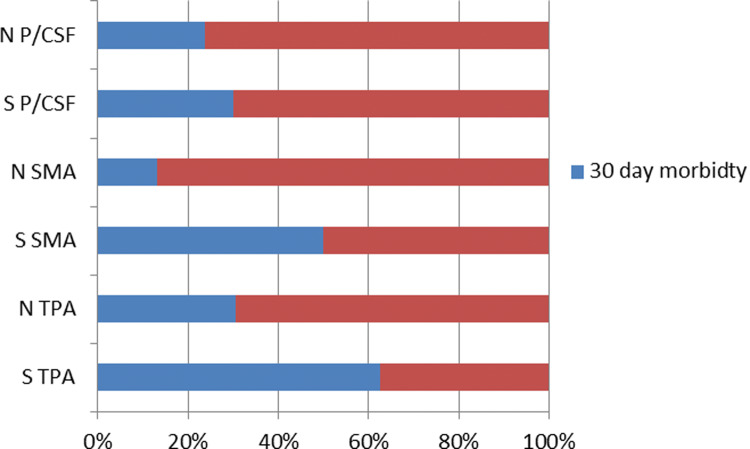
Morbidity in patients with and without MRE defined sarcopenia according to the measurements of psoas muscle intensity to cerebrospinal fluid intensity ratio (P/CSF), skeletal muscle area (SMA) and total psoas area (TPA). *S* value indicated sarcopenia, *N* normal range

In the group of 10 patients who suffered from postoperative complications the prevalence of MRE defined sarcopenia was 50%, 40% and 30% according the defined cut-off values of TPA, SMA and psoas intensity to CSF ratio, compared to 23.8%, 19% and 23.8% in the group of 21 patients who did not develop postoperative complications (Fig. 6).

### Inter observer agreement

There was high inter-observer agreement between the measurements of TPA and SMA recorded by the radiologist and by the surgeon, with ICC of 0.98 and 0.97, respectively, similarly to the agreement with the measurements reported by the radiographer (ICC 0.9 and 0.96 for TPA and SMA respectively). The ICC value between radiologist and surgeon for psoas muscle intensity to CSF ratio was 0.7, while with the radiographer was 0.83.

## Discussion

This pilot study demonstrates that MRE based cross-sectional measurements of the psoas muscle area and of the skeletal muscle area can be easily performed with high inter-observer agreement using conventional sequences obtained for the preoperative mapping of CD. We used these measurements as a surrogate marker of sarcopenia and found a higher rate of postoperative complications in the group of patients whose measurements were in the lowest quartile. Our study shows that MRE based measurement of the TPA and SMA is feasible and can be rapidly estimated not only by radiologists but also by trained surgeons, with an average additional time of 3 to 5 min required for MRE reporting.

Previous studies on imaging defined sarcopenia have focused mainly on the outcomes of trauma, emergency surgery and cancer surgery particularly in the elderly population given strong correlation between loss of lean muscle mass, frailty and old age [30]. It has been shown that sarcopenia assessed by measurement on CT is associated with increased post emergency surgery mortality and a recent meta-analysis [31] suggests that sarcopenia carries a significant increased risk of major complications after gastrointestinal tumour resection. The role of radiologically defined sarcopenia in predicting postoperative outcomes in IBD patients remains a growing topic, with the validity of existing data on the correlation between sarcopenia and surgical outcomes following IBD surgery remaining limited. A systematic review by Ryan et al. [32] including five observational studies of IBD patients suggested that sarcopenia correlates with an increased rate of major postoperative complications, which concurs with the results of our study. Most studies on radiologically measured sarcopenia in CD have utilised CT imaging due to its better accessibility, and it is a merit of our study to have assessed sarcopenia on MRI which is radiation free and can also be used interchangeably with CT for assessment of skeletal muscle area as there is strong correlation between CT and T2-weighted MRI measurements [33].

Sarcopenia occurs in people of normal or even elevated BMI [34] as well as in those who are underweight, highlighting the need for a formal assessment of sarcopenia in all CD patients, not just those who are visibly malnourished [35]. The cut-off values for MRE defined sarcopenia we computed in our specific CD population in this pilot need to be validated in a large prospective study with the aim of providing additional information to the MDT when planning surgical treatment of CD. Preoperative stratification of perioperative risk in patients with CD may improve clinical decision-making, including the need for preoperative optimisation and prehabilitation, intraoperative strategy (anastomosis versus stoma) and the specifics of postoperative care such as admission to the intensive care unit or postoperative nutritional support. Artificial Intelligence may facilitate automated assessment of imaging protocols in order to routinely include evaluation of the psoas muscle and abdominal wall muscles in all CD patients who have imaging, to add these surrogate markers of sarcopenia into the acquired data set.

Early detection of sarcopenia in patients with IBD is important to prevent undesirable outcomes [36]. However, we do not believe that the incidence of postoperative complication will linearly reflect the presence of preoperative sarcopenia as many other factors have been demonstrated to affect surgical outcomes, such as preoperative anaemia [37], immunosuppression and penetrating or recurrent CD. On the other hand, measures such as albumin, age and BMI alone are not reliable correlates of muscle mass [38]. Recent evidence suggests that psoas muscle cross sectional area is a practical surrogate measure of sarcopenia that can be readily and reliably measured by clinicians using standard image viewing software [21], as confirmed by our findings. A recent study [39], identified CT and MRI measurement of the skeletal mass as reliable assessments of sarcopenia in CD surgery in a cohort of 230 patients, however although all CD operations were included only major postoperative complications were reported, whilst our study reported on all postoperative outcomes in the specific group of patients with CD undergoing primary ileocaecal resection.

The definition of sarcopenia embraces not only lean muscle mass loss, but also strength and function [40] with a previous review [41] demonstrating considerable heterogeneity in the assessment of sarcopenia in the IBD population, likely explained by published studies having a small sample size or inconsistency in both nomenclature and techniques used to measure body composition [42, 43]. Our study was retrospective and had limited power which may explain the lack of statistical significance in the association between sarcopenia and postoperative complications, similarly to other studies [44]. Nonetheless it suggests an association between “MRI-defined” sarcopenia and postoperative complications in CD. Larger prospective studies are needed but hold the prospect that cross-sectional muscle assessment at MRE could supplant conventional sarcopenia assessment as a practical guide to perioperative management in CD.

Previous research has evaluated the role of preoperative imaging in the assessment of mesenteric adiposity, with abundant visceral adipose tissue having been associated with clinical recurrence [45] supporting the idea of a pathogenic role of the mesenteric fat in CD [46]. No recommendation can be made based on the results of our study, on the preferred modality to be adopted for MRE assessments of sarcopenia due to the small number of patients. Nevertheless the TPA measurement showed a favourable profile due to the rapidity of acquisition and analysis compared to SMA and the higher inter-observer agreement compared to psoas muscle intensity to CSF ratio. The assessment for myosteatosis at MRE was not immediate and might even require different sequences that we could not implement due to the retrospective nature of the study. In our IBD Unit we are currently developing a prospective study with focus on MRE defined sarcopenia and functional assessment of muscle function, also including a psoas muscle assessment at intestinal USS as part of an experimental pre and intraoperative dedicated USS protocol. [47].

## Conclusion

Psoas muscle cross sectional area and skeletal mass area can be estimated on Magnetic Resonance Enterography as surrogate markers of sarcopenia with high inter-observer agreement.
